# Development of Virtual Resource Based IoT Proxy for Bridging Heterogeneous Web Services in IoT Networks

**DOI:** 10.3390/s18061721

**Published:** 2018-05-26

**Authors:** Wenquan Jin, DoHyeun Kim

**Affiliations:** Department of Computer Engineering, Jeju National University, Jeju 63243, Korea; wenquan.jin@jejunu.ac.kr

**Keywords:** Internet of Things (IoT), proxy, web services, virtual resource (VR), Open Connectivity Foundation (OCF), resource directory (RD)

## Abstract

The Internet of Things is comprised of heterogeneous devices, applications, and platforms using multiple communication technologies to connect the Internet for providing seamless services ubiquitously. With the requirement of developing Internet of Things products, many protocols, program libraries, frameworks, and standard specifications have been proposed. Therefore, providing a consistent interface to access services from those environments is difficult. Moreover, bridging the existing web services to sensor and actuator networks is also important for providing Internet of Things services in various industry domains. In this paper, an Internet of Things proxy is proposed that is based on virtual resources to bridge heterogeneous web services from the Internet to the Internet of Things network. The proxy enables clients to have transparent access to Internet of Things devices and web services in the network. The proxy is comprised of server and client to forward messages for different communication environments using the virtual resources which include the server for the message sender and the client for the message receiver. We design the proxy for the Open Connectivity Foundation network where the virtual resources are discovered by the clients as Open Connectivity Foundation resources. The virtual resources represent the resources which expose services in the Internet by web service providers. Although the services are provided by web service providers from the Internet, the client can access services using the consistent communication protocol in the Open Connectivity Foundation network. For discovering the resources to access services, the client also uses the consistent discovery interface to discover the Open Connectivity Foundation devices and virtual resources.

## 1. Introduction

A wide range of the industries has been equipped with the massive and heterogeneous connected-devices through the growing technologies of the Internet of Things (IoT). The growing number of Internet-connected devices had surpassed the population of human more than 1.84 times in 2010 [1], and is expected to reach 50 billion in 2025 [2]. As a global network infrastructure, the IoT can be comprised of numerous connected-devices which are developed in multiple technologies to deploy in various industries such as transportation, healthcare, energy, smart home, etc. IoT devices can be equipped with embedded sensors, actuators, storages, processors, and communication modules to support functionalities in the network [3]. Based on the collaboration of IoT devices, the sensor and actuator networks can be ubiquitous to provide efficient, seamless, and comfortable services to end users through wired and wireless communications [4]. However, for deploying and developing heterogeneous IoT devices in those domain-specific or cross-domain IoT systems using various technologies, the specifications of frameworks, hardware platforms, and communication protocols need to be considered. Therefore, the diversity of IoT devices brings difficulty of deployment and development. Moreover, IoT devices must interwork with not only the newly deployed elements using emerging technologies but also the existing systems.

According to the environment where an IoT device is deployed, the IoT device can be directly connected to the Internet using a physical Ethernet, Wi-Fi radio, or cellular modem or through a proxy [5]. Many IoT devices equip with limited power supply, computing, and network platforms, and such constrained devices do not use pervasive network solutions [6]. Furthermore, many IoT-related protocols and standards have been published and applied for supporting management, discovery, and communication functionalities to IoT elements [7]. However, it is difficult to enable the connection between constrained networks and the Internet or different communication solutions because there is no a uniform standardization in communication protocols and network technologies and it is also not possible to support a uniform standard [8]. Moreover, traditional web services are provided based on the Hypertext Transfer Protocol (HTTP), it is not possible to be revised to match the IoT challenges, and it needs to consider many underlying IoT-specific protocols [9]. Therefore, the proxy is necessary for bridging the different elements in the IoT environment.

The proxy is an important element in the IoT network that supports protocol translation, registration, discovery, management, and other major functions [10]. The proxy is a necessary network element in the IoT which aims to enable communication between heterogeneous networks [11]. The perspective from service clients, the proxy bridges services including traditional web services and emerging IoT services from various domains through a consistent interface. For the appearance of services in the network where the proxy is deployed, the information of services needs to be registered. Especially, the IoT devices in the constrained environment cannot always keep the client updated about the device status because these devices are in a hibernate state most of the time [12]. The resource directory (RD) is used for registering the resources information to provide the information to clients [13]. The proxy involves the functionalities of RD to provide the information of resources for enabling the client to lookup accessible services in the network. Once the resource information is retrieved by a client, then the client can access the service which is exposed by the resource. The accessing to the service can be direct if the client and server use the same protocol, or through the proxy for converting the protocol. Therefore, the client needs to be aware regarding the server for selecting a suitable way to generate the request message whether the server is a service provider or a proxy. However, supporting a consistent client scheme for enabling transparent access to the different network can allow users do not consider which client to be used.

In this paper, an IoT proxy is proposed that is used for bridging the web services from the Internet to the IoT network. Most of the existing web applications are developed in HTTP based servers to provide services in the Internet. Through accessing a service from the server of web service provider (WSP), the information can be delivered to the client. The proposed IoT proxy supports transparent access to the web services for the client in the IoT network such as the Open Connectivity Foundation (OCF) network. The client in the OCF network can access IoT devices directly through the OCF communication protocol. For accessing the web services from the OCF network, the client needs to request the proxy for forwarding the message to the destination server in the Internet. However, the proposed IoT proxy presents the resources of WSPs in OCF network using OCF resources, and the OCF client can request the OCF resources to access services which are provided by the WSPs. The OCF resource is a virtual resource (VR) that represents the concrete resource of WSP in the OCF network. The VR presents the resource information for the web service through the discovery interface by the RD function of IoT proxy. Once the VR is discovered by the IoT client, the client can request to the VR for accessing the concrete resource in the Internet. In the forwarding process, the message translator function of IoT proxy translates the messages between the IoT client and WSP.

The rest of the paper is structured as follows; Section 2 introduces the related works including the existing solutions of proxies for interconnecting different protocols and related terminologies regarding this paper. Section 3 introduces models of the proposed IoT proxy and IoT network, and the methodology of translating scheme using proxy based on the VR in the IoT network. Section 4 introduces the scenarios of registration, discovery, and service accessing the resource in the proposed IoT network. Section 5 introduces implementation detail and evaluation results regarding the IoT proxy in the OCF network. Finally, this paper is concluded in Section 6.

## 2. Related Works

The emerging IoT standardization aims to support lower entry for developing services and improve the interoperability of different entities for the better performance of services [14]. In order to reduce the manufacturing costs of IoT devices, many frameworks have been published for the standardization of architecture, interfaces, protocols, and services to enable the development. One of them, the IoTivity, is a framework that is sponsored by the OCF for implementing the OCF standard specifications. The OCF core specification includes architectures of functions, resource models, definitions of properties, communication schemes, and other functional extensions such as discovery, notification, and group communication [15]. The OCF as a framework for the IoT environment that is designed to run on various systems such as Linux, Windows, Android, iOS, and Arduino [16]. The IoTivity is an implementation of OCF that support the core functions in OCF framework. Therefore, the constrained application protocol (CoAP) is the mandatory protocol for the communication, which is a protocol for providing IoT services with RESTful APIs by the constrained devices [17,18]. The CoAP is a standard application protocol that is designed for REST architectural style to be the optimized alternative to the HTTP [19]. According to the structure of the CoAP and HTTP, the proxy can be designed to be based on mapping the request and response messages. However, once the client requests to the destination server through the proxy, the requests need to be generated based on the URI of proxy with the parameters for requesting the destination server.

To implement the proxy for different protocols and network architectures, the proxy needs to involve the technologies of both sides, e.g., if an HTTP client wants to access a CoAP server, then the proxy needs to involve the implementation of HTTP and CoAP. In this case, most of the implementations support an interface from the proxy for receiving the HTTP request and forwarding to the CoAP server. However, the URI of the interface shows the client requests to the proxy, e.g., https://p.example.com/hc/?target_uri=coap://s.example.com/light [20]. This URI structure requires the client to select a specific proxy for the CoAP resource after the discovery. In another case, the request involves the ID and other parameters for accessing the destination server [21]. Then, the proxy retrieves the information of the destination server through the repository using the ID to get required information.

The publish–subscribe (PS) model enables the constrained devises as the clients in the network to publish their data when desiring to save power using sleepy or scheduling schemes [22]. The status of devices can be stored and synchronized with the devices in the physical world. The clients can subscribe the status information from the PS server to receive notifications once the device publishes its status. Many IoT communication protocols have been presented with this model, such as HTTP [23], MQTT [24], and CoAP [25]. Using heterogeneous communication protocols for the PS model, the PS server needs to provide publish interfaces for the devices in different protocols. Then clients request the subscribe interface to get data from the PS server.

In the OCF network, the OCF devices can be requested directly by the OCF clients. However, the servers in the non-OCF network cannot be requested directly. The proxy functions as the middleware between the client and the destination server, which can forward the original message to the destination server after translating for the protocol of server. In this case, the proxy needs to include the implementation of both protocols, e.g., HTTP client with OCF server or CoAP client with HTTP server, etc. [26,27]. A specification of OCF presents an architecture of OCF device that includes the client, server, and translator to forwarding message between different network environments [28]. In another case, the proxy can be a part of the destination server. The proxy acts as a partial function attached above the server to translate the received request for mapping the native interface of resources in the framework such as oneM2M [29,30]. Moreover, the client can be implemented to support more than one protocol for requesting the servers through multiple protocols. For accessing the destination server, the client must know beforehand regarding to the proxy [31]. The proposed IoT proxy in this paper also is required to be known by the client. However, the client is not aware regarding whether the accessed server is a proxy or not. Therefore, the client accesses all services using a consistent communication protocol without using the URI of proxy with the parameters.

Using the proposed proxy scheme, which bridges web services to the OCF network from the Internet. The existing server applications are mainly built in HTTP, although the services are provided for the IoT applications such as smart homes, smart cities, and other smart environments with a group of devices [32]. Therefore, many frameworks try to support the interworking scheme between IoT network and HTTP based servers [33]. Furthermore, the WSPs are equipped with high-performance parts to process thousands of requests using massive data which are used for composing response messages, such as weather information. Most of the web services are developed for providing HTTP based APIs using XML or JSON data formats to carry the information in the response payload. In the implementation details of this paper, the proposed proxy bridges the weather information provider—Open Weather Map (OWM) from the OCF network [34]. The WSP provides several weather-related services in free. Each API requires one or more parameters for accessing the service. For the appearance in the OCF network, the service information needs to be registered. The profile of WSP can be described by a specific data format, such as JSON, XML, etc. There are also popular frameworks to provide a data structure for involving properties and its values to describe web services, such as RESTful API Modeling Language (RAML) [35] and Swagger [36]. The OCF also have been presented many descriptions of IoT devices using those frameworks [37]. In the proposed proxy, RAML data model is used that enables the detail information can be described such as the basic information of WSP including the base URI, resource URIs, requirement of requesting the resources of WSP, and data model of response message. A handler of HTTP resource can be assessed by the API with the method, and the service exposes the resource to the Internet. We define the weather service of OWM using RAML which include the basic information of OWM and a resource specification including relative URI, parameters, and response schema in JSON.

## 3. Proxy-Based IoT Architecture

### 3.1. IoT Architecture Based on IoT Proxy in OCF Network

In the proposed IoT network, the IoT clients can access the services which are discovered in the network. The services are exposed by the resources of servers which are physical devices in the same network with the IoT client [38]. For the consistent scheme to access the services provided by heterogeneous networks, the IoT proxy can be the bridge to expose the services into the network where the IoT client is involved.

**Figure 1:**
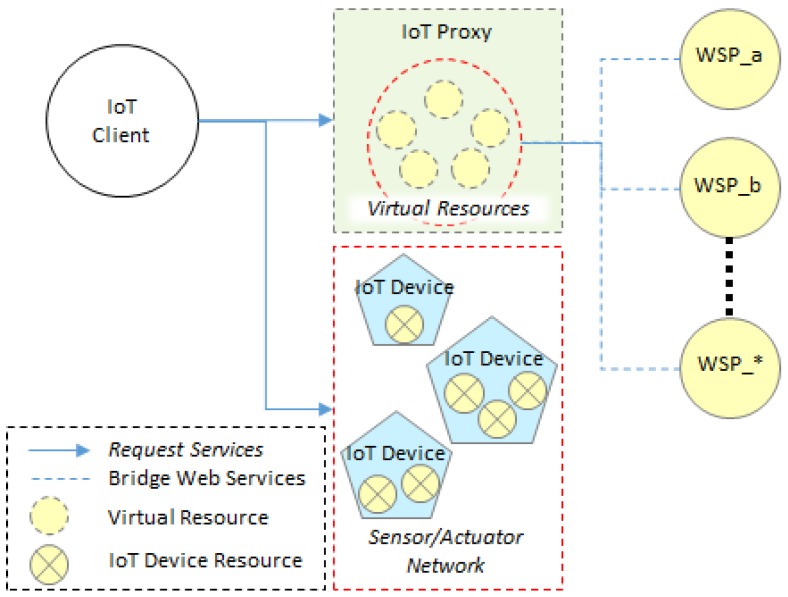
Proxy-based IoT architecture for providing services from sensor/actuator network and WSP.

Figure 1 illustrates the model of providing IoT services and web services to the IoT client in the proposed IoT network where the IoT proxy is deployed. The IoT client, IoT proxy, and IoT device are physical devices which communicate using a unique network protocol. The IoT services are provided by IoT devices and web services are provided by WSPs. Therefore, the resources of IoT devices and WSPs shall be enabled to access by the IoT client. An IoT device can include more than one resource to provide IoT services, and a sensor and actuator network can include more than one IoT devices. Those devices actually exist in the network where the IoT client is involved. The WSPs are deployed in the Internet, and servers of the WSPs provide services through HTTP in general. We assume the IoT network supports the OCF communications for the services. Then the IoT proxy can be the bridge for supporting the connection between IoT client and WSPs. Therefore, the IoT client enables access to services using a consistent protocol client such as OCF client in the OCF network. Through the IoT proxy, services of WSPs are exposed by the VRs of IoT proxy to the IoT client.

The VRs can appear to the IoT clients as the IoT resources which are included in the IoT devices. From the perspective of IoT client, the information of VRs for the WSPs and IoT resources for the sensor and actuator networks describe the same assessing interface because the IoT proxy and IoT device provide services through the same protocol. The request to the IoT device can be directly reached and handled by the handler of resource in the IoT device. The request to the WSP is handled first by the handler of VR for forwarding the request to the destination WSP. Once the WSP receives the request, then the WSP shall respond the result to IoT proxy, and IoT proxy responds the result to the IoT client. Therefore, the IoT client can access IoT devices and WSPs using consistent request client in this IoT architecture.

**Figure 2:**
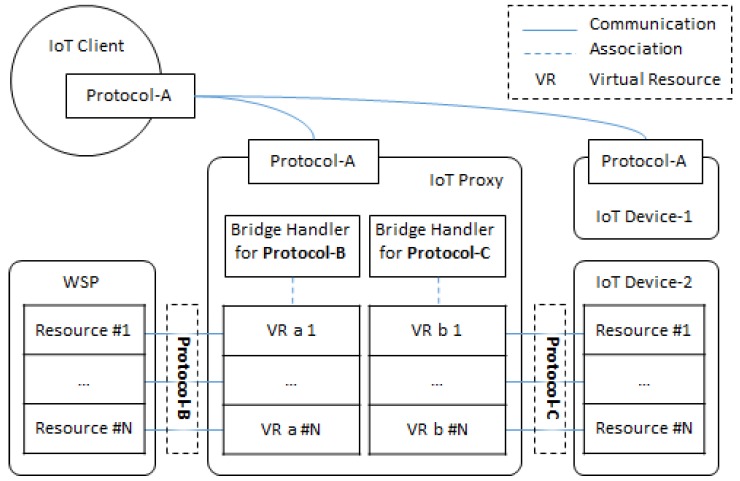
IoT network model based on proxy for bridging heterogeneous protocols.

Figure 2 shows the proxy-based IoT architecture. The architecture is comprised of IoT proxy, IoT device, WSP, and IoT client. The IoT proxy, IoT device, and WSP play the role of server to provide services to clients in the network. Those servers include resources to handle the service requests from clients. Each server entity can be structured to include perception layer, network layer, and application layer in the IoT environment for sensing the environment through sensors, connecting other devices to transmit data, and delivering services the users [39]. However, some devices on the Internet are not used for gathering information from the environment where the devices are deployed, rather they are used to service provisioning, e.g., the WSP in the proposed architecture.

The proxy-based IoT architecture involves heterogeneous entities with various protocols. We assume the IoT client is implemented to be based on the protocol-A in the network. Therefore, the IoT client only can request the servers which provide services through the protocol-A. Each entity has a protocol to be used for communicating with others which have the same protocol to be used. The WSP and the IoT device-2 provide services through protocol-B and protocol-C. In order to access those services, the IoT client needs the IoT proxy to forward the requests to the different protocols. For the different protocols, the IoT proxy implements different handlers which are used to instantiate the VRs. The functionalities of the handlers are used for translating and forwarding the message between different protocols. The VRs expose services to the IoT client through the same protocol with the IoT client. Therefore, the IoT client gets the results from the WSP and IoT device-2 through the exposed services from the IoT proxy.

**Figure 3:**
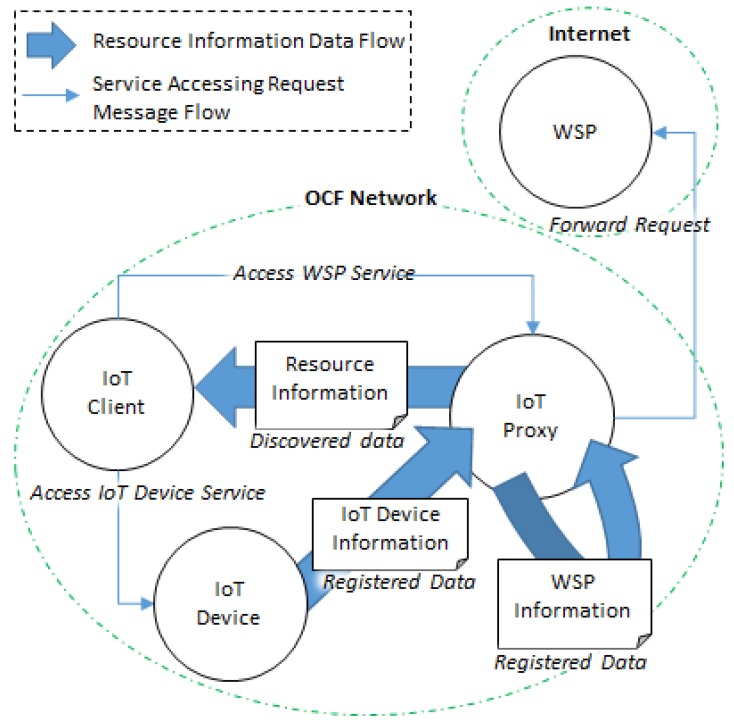
Interactions in the proposed IoT network model based on proxy.

For applying the proposed IoT proxy that is used for bridging the WSP in the Internet to the network where the IoT client is deployed, the OCF network is presented for deploying the IoT client, IoT device, and IoT proxy. Figure 3 shows interactions of IoT device information registration, WSP information registration, resource information discovery, IoT device service accessing, and WSP service accessing based on the OCF network and Internet. For the appearance of resources in the network, the resource information of IoT devices and WSPs need to be registered in an RD that provides discovery service to the IoT clients. The IoT device registers resource information by itself through publishing the IoT device information. The WSP cannot register resource information by itself because web services exist in the Internet. For registering the WSP resource information, the profile of the WSP can be made to register the information. The IoT client can discover the resource information from the RD through retrieving the list of registered resource information.

The IoT proxy includes the functionalities of RD for registration and discovery the information of resources, and as well as supporting the interworking proxy functionalities for bridging the IoT client to the WSP. For accessing services which are provided by WSPs, the IoT client sends the request message to the IoT proxy, and the IoT proxy forwards the request message to the destination WSP. For accessing services which are provided IoT devices, the IoT client sends the request message to the IoT device directly because the communication protocol is the same in both sides.

### 3.2. IoT Proxy Based on VR

**Figure 4:**
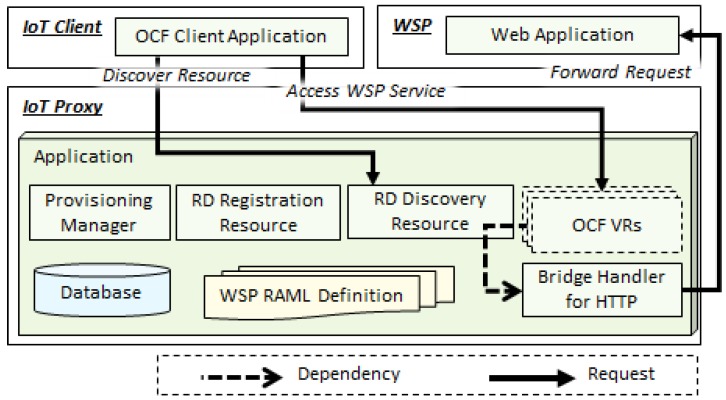
Proposed IoT proxy configuration based on VR.

The IoT proxy is a server as well as a client between the IoT client and WSP. The IoT proxy has the RD functionalities for registering information of resources which expose services in the network and discovering the information by IoT clients. The interworking proxy function of the IoT proxy is involved in the VR that translates the protocol to the destination protocol. Figure 4 shows the architecture of proposed IoT proxy in the OCF network for bridging the web services of WSPs in the Internet. The IoT proxy includes modules of the provisioning manager, RD registration resource, RD discovery resource, OCF VRs, bridge handler for HTTP, and database. The provisioning manager is used for initializing the network configuration and inserting information of WSPs through reading the RAML definition files. The information of WSPs is registered by the IoT proxy using the self-registration approach. Once the IoT proxy is stared, the application reads the files of RAML definition that involves resource information and WSP basic information. The application parses and inserts the information into the database for the registration process. The RD registration resource is used for providing the registration service to the IoT devices. The IoT devices register the resource information through requesting the resource of IoT proxy for being discovered by IoT clients.

The IoT client sends a request to the IoT proxy for discovering resources of WSPs and accessing the WSPs. The IoT proxy handles the discovering request through the RD discovery resource and handles the accessing request through the OCF VR. Once the RD discovery resource receives the request from the IoT client, the RD retrieves the information of resources from its database using the parameters of request message. The information is gotten from the RAML definition that is handled by the IoT proxy to insert into the database. The client gets the information of WSP that is included in the list of OCF resource information. Therefore, the WSP information is recognized as an OCF server. Then the IoT client can request the resource in the list using a consistent message sender. The OCF VR for the destination WSP that handles the service accessing request, and forwards the message to the WSP. The OCF VR is generated using the bridge handler that is an OCF resource handler for translating the messages between OCF based IoT clients and HTTP based WSPs.

### 3.3. Message Translator in IoT Proxy

An OCF VR shall be generated when a WSP resource information is read from a RAML file in the IoT proxy. We implement one OCF bridge handler for each bridged protocol. Therefore, for the WSP that provides the HTTP-based services in the Internet, a bridge handler shall be implemented for bridging the HTTP servers.

For generating an OCF VR, the IoT proxy gets the information from the WSP’s RAML definition to store in the database and uses the bridge handler entity and WSP information to generate the OCF resource. The handler of OCF resource refers the functions of bridge handler entity. Therefore, once the OCF VR is accessed, the handler of VR shall be triggered and runs the method of bridge handler entity.

**Figure 5:**
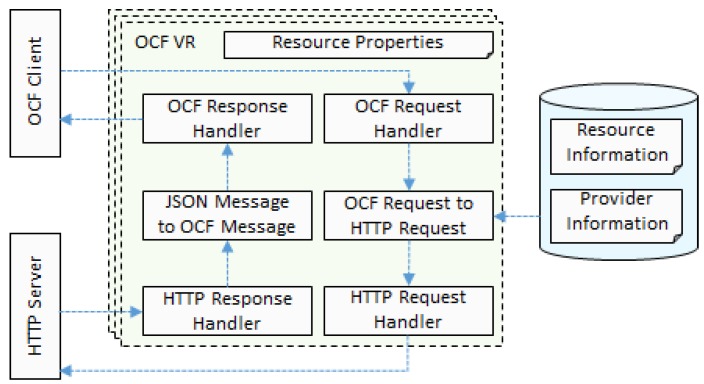
Message translating mechanism for OCF to HTTP.

The message translating mechanism is illustrated using the data flows with functional blocks in the Figure 5. The OCF client can discover the OCF VR in the OCF network. The OCF VR is an OCF resource that includes resource properties and OCF method handlers such as GET, POST, PUT, and DELETE. Each method handler refers to the instance of bridge handler for bridging the HTTP servers.

The flowing steps illustrate the changes of request and response messages in the translating data flow for accessing a service of WSP from the OCF client.

1. The OCF client requests the OCF VR, then the OCF request handler receives the message.
2. The OCF-request-to-HTTP-request function gets the query parameters from the request message, and gets WSP’s URI from the database. Using that information, the function generates an HTTP request message for accessing the service of WSP.
3. The HTTP request handler sends the HTTP message to the HTTP server of WSP.
4. The HTTP response handler receives the HTTP message from the HTTP server of WSP.
5. The JSON-message-to-OCF-message function gets the information from the JSON message that is sent from the HTTP server through the payload of response and generates the OCF message.
6. The OCF client receives the OCF response from the OCF response handler of OCF VR.

## 4. Registration, Discovery, Service Accessing of Web Service, and IoT Service

For implementing the proposed IoT architecture based on the IoT proxy, scenarios of registration, discovery, and service accessing are presented through interactions between entities in the IoT environment. The interaction of registration is used for registering the information of service entities which need to be accessed, i.e., web services in the Internet and IoT services from IoT devices in smart homes, factories, buildings, etc. The interaction of discovery is used for discovering the information of resources in the OCF network. The IoT client shall discover the resources using the discovery interaction, and access the services which are provided by resources of IoT devices and VRs of IoT proxy for the WSPs. The interaction of accessing service is used for accessing services which are provided by OCF servers in the network where the IoT client is included. The IoT client can access services from IoT devices which provide indoor services in smart homes, hospitals, factories, and other smart indoor spaces. Through the IoT proxy, the IoT client can also access services from WSPs which provide outdoor services such as information of weather, traffic, and natural disaster.

**Figure 6:**
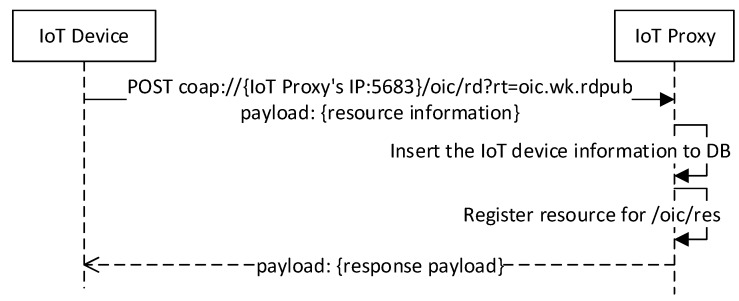
IoT device information registration.

Figure 6 illustrates the sequence diagram for registering an IoT device information. The IoT device sends the request message using POST method to the IoT proxy. The RD of IoT proxy receives the request, inserts the information to the database, and registers resource for enabling discovery from /oic/res resource. The request URI structure includes the URI of IoT proxy with the relative URI /oic/rd, and the query parameter for the resource type of RD. The request payload includes the information of resources which are implemented in the IoT device. The response payload includes the registered resources information.

**Figure 7:**
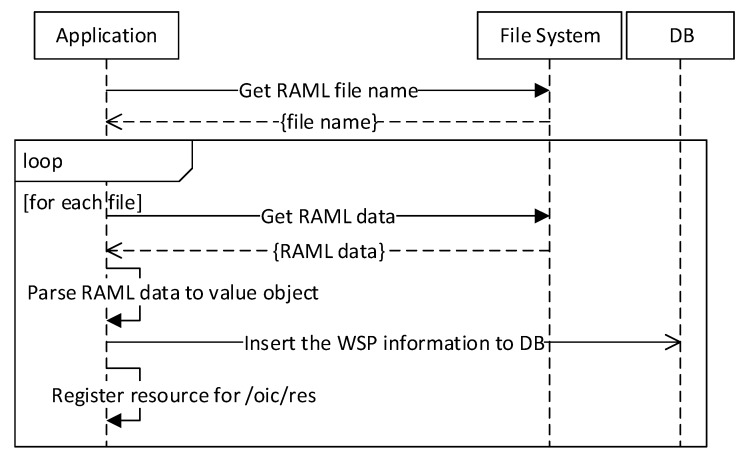
WSP information registration.

Figure 7 illustrates the sequence diagram for registering a WSP information. The information is involved in a RAML file that is deployed in the IoT proxy. The IoT proxy reads the RAML file and gets the information of resources. If there is more than one available RAML file, then the process of getting resources information shall be called again and again until all files are read. In the IoT proxy, the application gets the RAML data from the RAML file that is formatted in a RAML definition. The parser function gets the information from the RAML data and puts to value objects. Using the value objects the application inserts the WSP information to the database and registers resource for enabling discovery from /oic/res resource.

**Figure 8:**
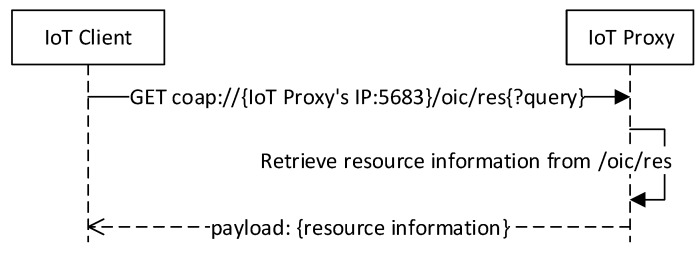
Resource discovery based on IoT proxy.

Figure 8 illustrates the sequence diagram for resource discovery in the OCF network. The OCF resources expose services in the network, and for accessing the services, the client needs to know the information of OCF resources. Through the registration process, the IoT proxy has the information of IoT devices and WSPs. The OCF resources of IoT devices and VRs for WSPs are available to be discovered through the resource /oic/res in the IoT proxy. The request to the resource /oic/res can be structured with the URI of IoT proxy, relative URI /oic/res, and query parameters. The queries can be resource types and resource interfaces which follows the specification of OCF standard. The response payload includes the list of resource information that is formatted in the OCF proposed specification.

**Figure 9:**
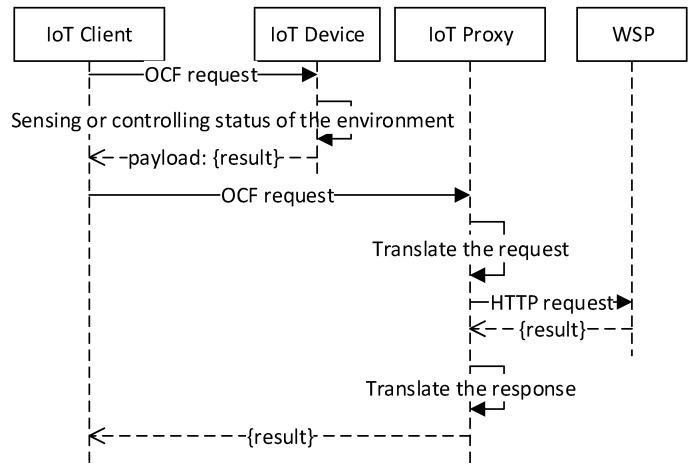
Transparent access to services of IoT device and WSP.

Figure 9 illustrates the sequence diagram for accessing a transparent service that is provided by an IoT device, and a service that is provided by a WSP through the IoT proxy. The IoT device can provide the indoor services using the sensors and actuators. Through the VRs, the IoT proxy can provide outdoor services which are provided by the WSP. The IoT device resources and VRs represent the information and functions of parts which are discovered by the IoT client. Through the discovered information, the IoT client sends the OCF request to the IoT device and IoT proxy. Once the IoT device receives the request, then the handler of the resource gathers the sensing data or controls the actuator in the indoor environment. Once the IoT proxy receives the request, then the handler of the VR translates the OCF request to the HTTP request and sends to the WSP. The response from the WSP also needs to be translated, and the IoT proxy returns the result to the IoT client.

## 5. Performance Analysis

### 5.1. Implementation of IoT Network Based on Proxy

In this experimental environment, the services of IoT devices can be indoor services in the home network, and the services of WSPs can be outdoor services from the Internet. The IoT proxy bridges IoT client and WSP which provides services based on HTTP. In the perspective of IoT client, indoor services and outdoor services are provided by the OCF server, because the services of WSP are available in the OCF network through the IoT proxy. The indoor service is registered via sending the information of IoT device using POST /oic/rd request to the IoT proxy by the IoT device and the outdoor service is registered by the IoT proxy itself. Then the IoT client can discover indoor and outdoor services in the OCF network using GET /oic/res request to the IoT proxy. Once the information of resources is retrieved by the IoT client, the user can access the services of IoT device and WSP.

**Figure 10:**
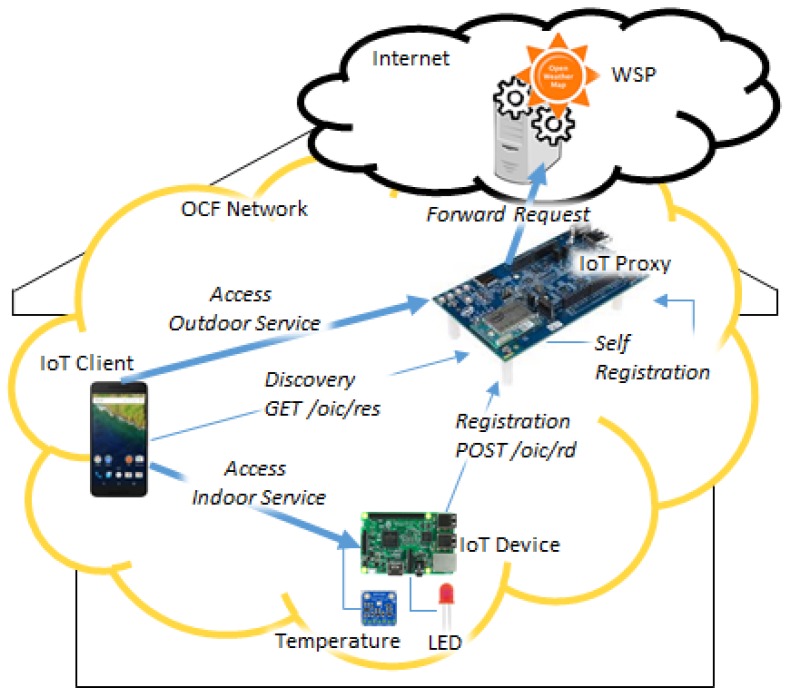
Experimental environment.

Figure 10 shows the experimental environment which is comprised of the OCF network and the Internet. The OCF network can be a home network to support the connectivity for the devices through the LAN such as Wi-Fi networking which is provided by a Wi-Fi router. The OCF network includes IoT client, IoT proxy, and IoT device, which communicate with other entities through the OCF protocol in the LAN. In the Internet, the WSP is a weather service provider which provides weather data in the JSON format through the APIs. In the implementation of the OCF network, the IoT client is an Android phone which supports the UI to interact with the users; the IoT proxy is an IoT board which enables the IoT client to communicate with the WSP using the Wi-Fi connection; the IoT device is an IoT board which equips a temperature sensor and a LED, provides the temperature sensing service and the LED controlling service, and also communicates with the IoT client using the Wi-Fi connection.

Table 1 illustrates the development environment of the entities in the experimental environment. The application development tool is the Android Studio 3.0.1, it also is used for the analysis of performance. The Android OS runs on those entities of the OCF network, therefore, Java is the language for implementing the applications. The detail specification of Android platforms is different for each entity, because the platform depends on the physical device.

The IoT proxy runs on Intel Edison Board which operates Android Things 0.2, and the application is supported minimum Android version is SDK 24, and it is developed using IoTivity 1.3.0 (×86) for OCF server, raml-parser-2 1.0.13 for RAML parser, and Volley 1.0.0 for HTTP client.

The IoT device runs on Raspberry Pi 3 Model B which equips a BMP 280 and a LED and operates Android Things 0.4.1, and the application is supported minimum Android version is SDK 24, and it is developed using IoTivity 1.3.0 (armeabi) for OCF server, com.google.android.things.contrib: driver-bmx280:0.4 for sensing the temperature.

The IoT client runs on Samsung Galaxy S4 and operates Android 5.0 Lollipop, and the application is supported at minimum in Android version SDK 21 and compiled in SDK 26, and it was developed using IoTivity 1.3.0 (armeabi) for OCF client.

For the WSP, an open weather API provider is selected which is Open Weather Map. Open Weather Map provides weather-related APIs based on JSON and XML data formats. In the implementation, the service is used to provide the current weather information of a location. The resource of service is defined in the RAML which is deployed in the IoT proxy for registering the WSP. In the RAML definition, baseUri is http://api.openweathermap.org/data/2.5, the resource name is /weather, queryParameters are if for the OCF resource interface, q for the query of location which is required for accessing the API, and APPID for the API access key which is also required for accessing the API.

**Figure 11:**
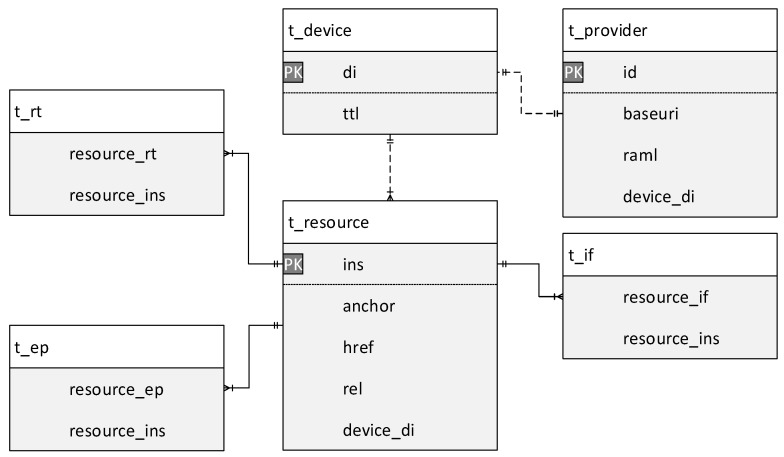
IoT proxy ER-diagram.

Figure 11 shows the ER-diagram for the database (DB) of IoT proxy. In order to store the information of IoT device and WSP in the proposed system, a DB in the IoT proxy is presented. The information which is stored in the DB is presented to be mainly divided into two categories. The resource information is stored in the tables, i.e., t_device, t_resource, t_rt, t_if, and t_ep. The provider information is stored in the table t_provider.

Through the following example information of IoT device and WSP, the usage of each table is explained.

Table t_device is used for storing device ID and retention time. The UUID is used for the device ID of IoT device and WSP. The IoTivity framework generates the UUID for the entity in the initial process. Once the IoT proxy is started, the UUID is generated for each WSP information.

Table t_resource, t_rt, t_if, and t_ep are used for storing the OCF resource properties which can be referred to the specification of OCF. The values of anchor, href, rel, rt, if, and ep are attributes of an OCF entity which is generated once the OCF entity is initialized. For the registering of an IoT device, the IoTivity framework also supports packing of those values to a JSON data. Because the registration specification in OCF network requires a standard message structure.

Table t_provider is used for storing the non-OCF related information of WSP. The baseUri is an attribute of RAML definition that is the basic URI of the service provider. The attribute baseuri in the table that stores the basic URI of WSP. For the weather API of Open Weather Map, the baseUri can be http://api.openweathermap.org/data/2.5 and the full URI which includes the resource name can be http://api.openweathermap.org/data/2.5/weather.

The data format and contents for publishing information of an IoT device to the IoT proxy follow the OCF specifications. Once the IoT proxy receives the registering message, the information of message shall be inserted to the proposed tables in the database. The WSP information also shall be inserted to the database by the self-registration process. The RAML definition is used for involving the information of a WSP.

The data format and contents for publishing information of IoT device to the IoT proxy that follows the OCF specification. Once the IoT proxy receives the registering message, the information of message shall be inserted to the proposed tables in the database. The WSP information also shall be inserted to the database by the self-registration process. The RAML definition is used for involving the information of a WSP.

**Figure 12:**
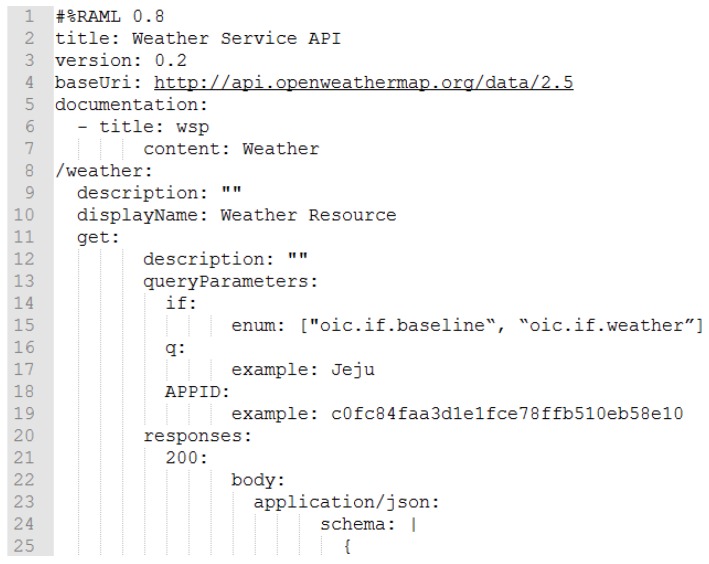
Fragment of RAML definition for registering WSP.

Figure 12 shows a fragment of RAML definition for registering a WSP information. The service in the Internet can be described by the RAML definition, and the information can be presented to the client by the service of RD. The information of IoT devices can be registered by the devices, however, the WSPs cannot register the information by the providers except they include the publishing function in their system. The RAML definition includes a service that exposed by the resource/weather. The provider’s base URI is http://api.openweathermap.org/data/2.5 that shall be used with other relative URIs for requesting services from the WSP. The resource/weather requires parameters if, q, and APPID, and the response message is defined in JSON schema in the fragment. The value of if must be the resource interface that is used for the OCF purpose. The value of q must be the location name, and APPID must be the key for requesting the service.

**Figure 13:**
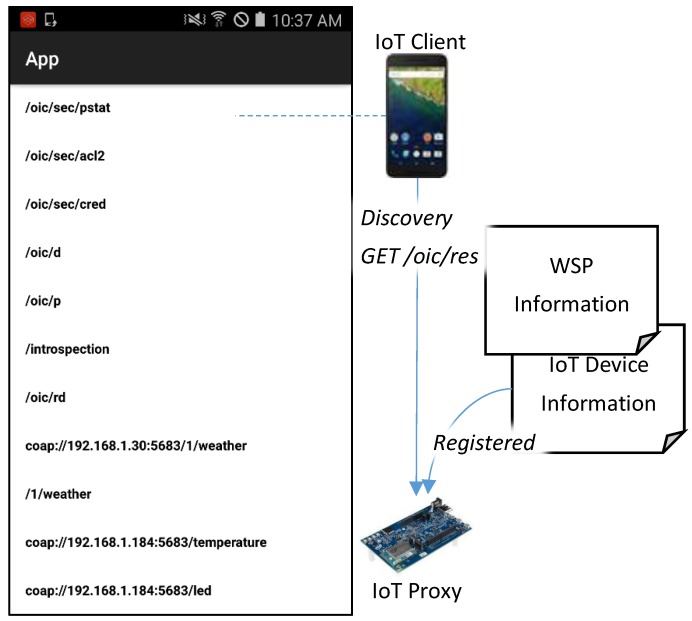
Fragment of RAML definition for registering WSP.

Figure 13 presents the implementation result of discovering the resource information. The screenshot shows the list of resource information which is discovered by the IoT client. For the discovery, the IoT client requests the resource information from the IoT proxy using GET/oic/res request. The IoT device information and WSP information are registered to the IoT proxy, which is retrieved with the resource information of the IoT proxy together. In the list, the URIs from IoT device are coap://192.168.1.184:5683/temperature and coap://192.168.1.184:5683/led, which can be accessed by the OCF client directly. For the WSP, the URIs coap://192.168.1.30:5683/1/weather and /1/weather, but the /1/weather is used for implementation that can be ignored. The user of theIoT client can click an item from the list to the service accessing page.

**Figure 14:**
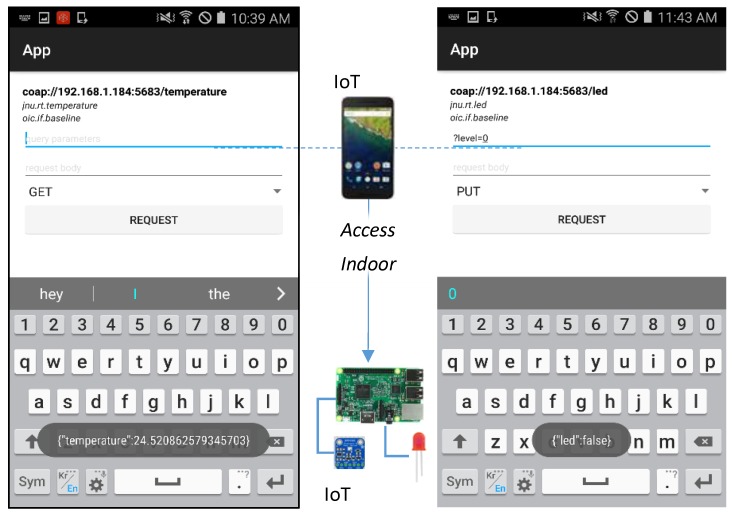
Fragment of RAML definition for registering WSP.

Figure 14 presents the implementation result of accessing the indoor service from the IoT device. The IoT device equips a BMP 280 and a LED for the temperature sensing service and LED controlling service. The screenshots of IoT client show the service accessing pages for gathering the temperature data and controlling the LED. For accessing the temperature sensing service, the GET method needs to be selected. Then the user can click the REQUEST button to get the current temperature value of the sensor. For accessing the LED controlling service, the query parameter level is required and the PUT method needs to be selected. Once the temperature sensing service is requested then a temperature value in JSON format is returned, and the LED controlling service is requested then the status of LED in JSON format is returned.

**Figure 15:**
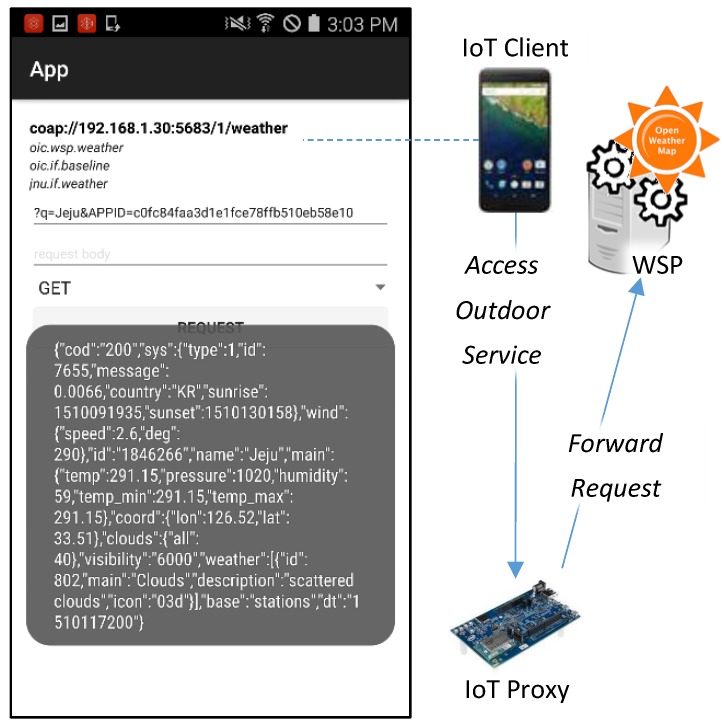
Fragment of RAML definition for registering WSP.

Figure 15 presents the implementation result of accessing the outdoor service from the WSP. The outdoor service is a service that provides weather information including temperature, wind speed, humidity, etc. The service is provided by the WSP that is the free weather provider—Open Weather Map. The WSP provides the weather information according to a location parameter. For the request to the WSP, the user needs to input the query parameter on the IoT client for the location. In the service accessing page, the IoT client shows the OCF resource information for the VR that bridges to the WSP. The information presents VR’s URI, and other OCF resource properties. For accessing the weather service, the parameters q and APPID are required. The parameter q requires a location, and Jeju is inputted. The parameter APPID is API access key, and the key is got from Open Weather Map for accessing APIs from the provider. Once the REQUEST button is clicked, the request message shall be delivered to the WSP, and the response message shall be returned to the client. Then the client shall display the result as shown in the page.

### 5.2. Performance Evaluation

**Figure 16:**
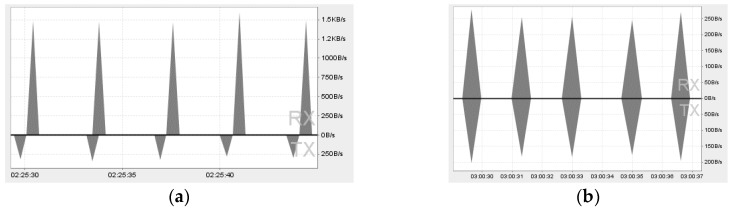

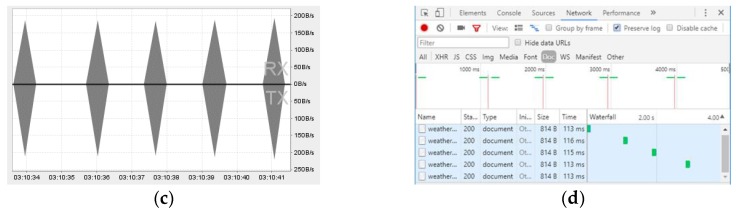
Network monitoring for accessing services. (**a**) Interaction A; (**b**) Interaction B; (**c**) Interaction C; (**d**) Interaction D.

Figure 16 shows the network monitoring for accessing services of the IoT device and the WSP. For collecting data to compare the size of data packets and round trip time (RTT) in the communications with elements of the IoT proxy based OCF network, four types of service accessing interactions are presented and each interaction is executed five times. The presented interactions are introduced as follows.

Interaction A: This interaction presents accessing the WSP’s service using the IoT client through the IoT proxy: This interaction is between the IoT client and the WSP through the IoT proxy which works on the OCF network between the IoT client and the IoT proxy, on the Internet between the IoT proxy and the WSP. The figure of Interaction A is captured from network monitor of the IoT client which shows the communication specification of the request from the IoT client to the IoT proxy. The maximum network speed of request message transmission is 250 B/s approximately and the maximum network speed of response message transmission is 1.5 KB/s approximately, and the range of RTTs are between 979 ms to 1324 ms.

Interaction B: This interaction presents accessing the temperature sensing service of IoT device using the IoT client: This interaction is between the IoT client and the IoT device directly which works on the OCF network between the IoT client and the IoT device. The figure of Interaction B is captured from network monitor of the IoT client which shows the communication specification of the request from the IoT client to the IoT device. The maximum network speed of request message transmission is 200 B/s approximately and the maximum network speed of response message transmission is 250 B/s approximately, and the range of RTTs are between 632 ms and 686 ms.

Interaction C: This interaction presents accessing the LED controlling service of the IoT device using the IoT client. This interaction is same as Interaction B except for the API specification and the figure of Interaction B is also captured by the same way with Interaction B. The maximum network speed of request message transmission is 200 B/s approximately and the maximum network speed of response message transmission is 200 B/s approximately, and the range of RTTs are between 685 ms to 649 ms.

Interaction D: This interaction presents accessing the WSP service using the web browser. This interaction is between the IoT client and the WSP directly which works on the Internet between the web browser and the WSP. The figure of Interaction D is captured from network monitor of the web browser which shows the communication specification of the request from the web browser to the WSP. The response message size is 814 B and it depends on the data which is delivered by the service of the WSP. Therefore, for accessing the service, the response message sizes are same. The range of RTTs is between 113 ms to 116 ms.

Interactions A, B, and C are monitored by Android Device Monitor of Android Studio 3.0.1. The monitored information shows network speed, transmission time, and packet size of RX (receive) and TX (transmit).

Interaction D is monitored from the development tool of Firefox. The monitored information shows transmission time, packet size of response message, and other network elements of HTTP communication.

**Figure 17:**
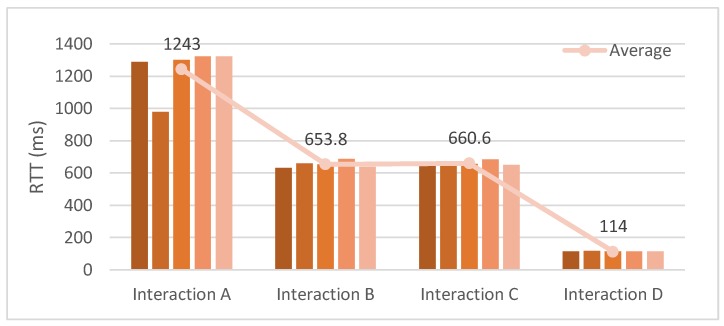
Comparison of RTTs for accessing services.

Figure 17 shows the comparison of RTTs for accessing services via Interaction A, B, C, and D. The average RTT of Interaction A is 1243 ms which is the highest of them because it is comprised of two request/response processes via the OCF network and the Internet. The average RTT of Interaction D is 114 ms which is the lowest of them. The average RTT of Interaction B is 653.8 ms and Interaction C is 660.6 ms, which are almost same because those interactions work on the same network environment and the processes of temperature sensing and the LED controlling in the IoT device proceed almost immediately.

Interaction D is monitored from the development tool of Firefox. The monitored information shows transmission time, packet size of response message, and other network elements of HTTP communication.

Table 2 shows the network packet sizes of Interaction A, B, C, and D. For Interaction A, the IoT proxy totally receives 975 B from the IoT client and the WSP, transmits 834 B to the IoT client and the WSP. Only for the interaction of the IoT proxy and the WSP in Interaction A, the IoT proxy receives 866 B from the WSP, transmits 335 to the WSP. The IoT client receives 499 B from the IoT proxy, transmits 109 B to the IoT proxy for Interaction A. So, the request message size from the IoT client to the IoT proxy is 109 B and the response message size is 499 B for accessing the service of the WSP. However, the IoT proxy receives 866 B from the WSP because the IoT proxy converts the HTTP message to the OCF message. For Interaction D, the web browser receives 814 B, and only for content is 456 B. Therefore, the interaction using the IoT proxy saves 415 B for accessing the service of the WSP from the IoT client.

**Figure 18:**
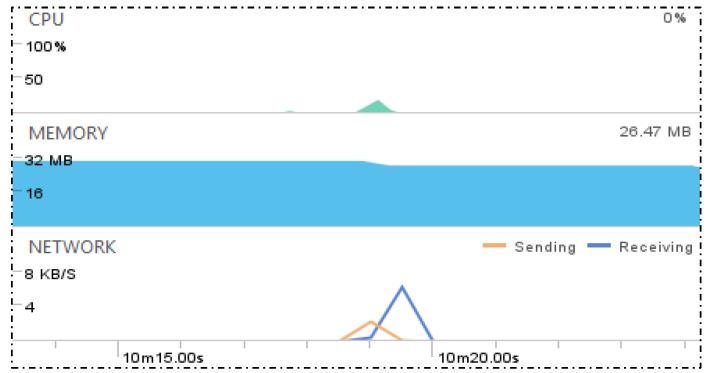
Status of IoT proxy for accessing a WSP service through IoT proxy.

Figure 18 shows the status of the IoT proxy for accessing the WSP service through the IoT proxy. The status is monitored for CPU usage, memory usage, and network speed by the tool of Android Studio 3.0.1. At the moment of accessing the service of the WSP from the IoT client, the handler of the VR in the IoT proxy is triggered to process the task. In this task which is monitored, the maximum CPU usage is 19.75%, maximum memory usage is 30.75 MB, and maximum network speed is 6.16 KB/s.

**Figure 19:**
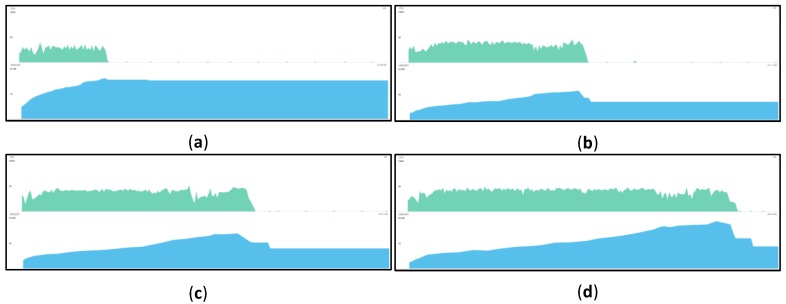
CPU and memory monitoring for registering WSP information. (**a**) Register 1 WSP Information; (**b**) Register 4 WSP Information; (**c**) Register 6 WSP Information; (**d**) Register 9 WSP Information

Figure 19 shows the usage of CPU and memory in the IoT proxy for registering the WSP information. We had deployed the RAML files in the IoT proxy for registering the WSP information. The experiment proceeded by deploying 1 to 10 RAML files in the IoT proxy. The registering of the WSP information using 1, 4, 6, 9 RAML files was selected to be shown, and the differences are obviously displayed for the usage of CPU and memory. In each monitoring figure, the top half is the usage CPU and the bottom half is the usage of memory. The IoT proxy application consumes the CPU in the process of registering the WSP information and the usage of memory is increased until the registration is finished. After the registration process is finished, the usage of CPU becomes 0% and the usage of memory become 25~30MB approximately, except for outliers. As shown in the differences of the figures, the cost of CPU and memory are increased with the number of the registered WSP information.

The initial memory usages are approximately same for each process and the range of sizes are between 6.64 MB and 10.47 MB in this experiment. The registering process runs on the start of the application, and the initial memory usages can illustrate the total size for external files, configuration files, resource files, and class instances from the Android application. The maximum memory usages and the registering times are increased with the number of the registered WSP information. The RAML parser in the Android application of IoT proxy may lock the thread of the process. Because the process runs on a thread, it means the Android application proceeds processes in same time for each registering of the WSP information. However, the registering times are still increased, and it is supposed because of the RAML parser. Therefore, if the RAML parser can run for multi-threads then the registering times are possibly not increased.

## 6. Conclusions

In this paper, the IoT proxy has been presented using VRs to bridge WSPs from the Internet to the OCF network. The proposed IoT proxy enables the IoT client having the transparent access to IoT devices and WSPs through the consistent service accessing scheme. Moreover, the VRs and resources of IoT devices are discovered together by the client as OCF resources through the discovery service that is provided IoT proxy. For the proposal of proxy based scheme, the architecture of IoT network and message translating methodology have been presented for the interworking of heterogeneous protocols. For developing the proposed IoT proxy in the OCF network, the scenarios of registration, discovery, and accessing of services, and presented the implementation details have been presented for indoor and outdoor services using the IoT device and the concrete web service that is provided via the OWM through the open API. Moreover, according to the evaluation results, the IoT proxy can reduce the size of the message for the delivered service from the Internet to the IoT client. The payload of response message is provided by the HTTP server to the IoT client through the IoT proxy. Therefore, the message translator of IoT proxy gets the JSON based payload data from HTTP response message to generate the OCF response for the client in the IoT network.

## Figures and Tables

**Table 1:** Implementation environment

Entity	H/W	Platform	Framework and Library
IoT Proxy	Intel Edison Board	Android Things 0.2 (Android min SDK:24)	IoTivity 1.3.0 (×86), raml-parser-2 1.0.13, Volley 1.0.0
IoT Device	Raspberry Pi 3 Model B, BMP280, LED	Android Things 0.4.1 (Android min SDK:24)	IoTivity 1.3.0 (armeabi), com.google.android.things.contrib:driver-bmx280:0.4
IoT Client	Samsung Galaxy S4	Android 5.0 Lollipop (Build: compile SDK 26, min SDK 21)	IoTivity 1.3.0 (armeabi)

**Table 2:** Network packet sizes of interactions

Element	Interaction	RX Size	TX Size
IoT Proxy	Interaction A	975 B	834 B
Bridge Handler	Interaction A	866 B	335 B
IoT Client	Interaction A	499 B	109 B
IoT Client	Interaction B	88 B	64 B
IoT Client	Interaction C	64 B	73 B
Web Browser	Interaction D	814 B	-
Content	Interaction D	456 B	-
